# Prevalence of anemia and iron deficiency anemia in Chinese pregnant women (IRON WOMEN): a national cross-sectional survey

**DOI:** 10.1186/s12884-020-03359-z

**Published:** 2020-11-07

**Authors:** Jing Tan, Guolin He, Yana Qi, Hongmei Yang, Yiquan Xiong, Chunrong Liu, Wen Wang, Kang Zou, Andy H. Lee, Xin Sun, Xinghui Liu

**Affiliations:** 1 Chinese Evidence-based Medicine Center, West China Hospital, Sichuan University, No.37 Guo Xue Xiang, Chengdu, China; 2 Department of Obstetrics and Gynecology, and Key Laboratory of Birth Defects and Related Diseases of Women and Children (Sichuan University), Ministry of Education; West China Second University Hospital, Sichuan University, No.37 Guo Xue Xiang, Chengdu, China; 3 School of Public Health, Curtin University, Perth, WA Australia

**Keywords:** Anemia, Iron deficiency anemia, Prevalence, Risk factors, Cross-sectional survey

## Abstract

**Background:**

The current evidence about anemia and iron deficiency anemia (IDA) during pregnancy remains elusive in China. The purpose of this study is to investigate the prevalence of anemia and IDA and their risk factors in Chinese pregnant women.

**Methods:**

A nationwide cross-sectional survey of pregnant women was conducted during their antenatal visits. Using a multi-stage sampling method, 24 hospitals from 16 provinces across China were selected. Structured questionnaires were administered to collect information from participants and to extract clinical data from electronic medical records. Mixed-effects logistic regression models were performed to determine the risk factors associated with anemia and IDA.

**Results:**

In total, 12,403 pregnant women were enrolled, including 1018 (8.2%) at the first trimester, 3487 (28.1%) at the second, and 7898 (63.7%) at the third. Overall, 19.8% of women were diagnosed with anemia and 13.9% were diagnosed with IDA. The prevalence of anemia and IDA varied among regions and increased by gestational month, peaking at the eighth gestational month (24.0% for anemia and 17.8% for IDA). Pregnant women at advanced stage of gestation, non-local residents, multiple gestations, multiparity, pre-pregnancy underweight, and those experiencing severe nausea or vomiting during pregnancy, were associated with higher risks of anemia and IDA.

**Conclusions:**

The prevalence of anemia and IDA during pregnancy are similar to those from developed countries and vary across regions in China.

## Background

Anemia is the most common pregnancy comorbidity [1, 2]. A systematic review of evidence from 107 countries suggested that the prevalence of anemia among pregnant women was 38% (95% CI 33–43%), impacting approximately 32 million individuals [3], of whom about 75% were manifested with iron deficiency [4]. The prevalence of iron-deficient anemia (IDA), the major type of anemia, appears to vary across regions, from 3% in Europe [5] to over 50% in Africa [6, 7].

Anemia and IDA can lead to adverse health outcomes for both mothers and their offspring, including infections, premature rupture of membrane, fetal growth restriction, fetal hypoxia, premature birth, low birth weight and fetal death [8, 9]. In low- and middle-income countries, 12% of low birth weight, 19% of preterm births, and 18% of perinatal mortality, are attributable to maternal anemia [10].

In China, a large quantity of deliveries takes place every year.. In 2016, for example, there were 18,466,561 live births [11]. Given the recently implemented two-child policy, reducing anemia and IDA during pregnancy has become a national priority for antenatal care. However, the available latest prevalence data was outdated that was from a survey (*n* = 3591) conducted nearly two decades ago [12].

In the last decade, China has undergone dramatic social and economic reforms, which exert profound impacts on pregnancy care, lifestyles, and maternal and child health. The lack of up-to-date information on the prevalence and risk factors of anemia and IDA has presented a major obstacle for developing evidence-based recommendations for managing pregnant women. To address such an important knowledge gap, we conducted a nationwide cross-sectional survey to investigate the current prevalence of anemia and IDA during pregnancy. Using a hybrid data collection process, the risk factors associated with the development of anemia and IDA for Chinese women were also ascertained in this study.

## Methods

### Study design

From 19 September to 20 November 2016, a nationwide cross-sectional survey was conducted at 24 hospitals from 16 provinces across China. The hospitals were selected according to the multi-stage sampling method (detailed below). Pregnant women, who attended these hospitals for antenatal care, were consecutively recruited into the study regardless of their gestational age.

The study was approved by the Research Ethics Committees of West China Second Hospital, Sichuan University (2016–009), Tongji Medical College of Huazhong University of Science and Technology (2016-S159), Beijing Friendship Hospital, Capital Medical University (2016-P2–058-01) and The First Affiliated Hospital of Zhengzhou University (2016–26). It was registered at ClinicalTrials.gov (ID number: NCT02887963). We have obtained informed consents from all participants. The Department of Obstetrics at West China Second University Hospital and the Chinese Evidence-based Medicine Center at West China Hospital, both from Sichuan University, took the primary responsibilities for study planning and design, project management, data collection, statistical analysis, and reporting.

### Sampling methods

We used a multi-stage sampling method for selecting hospitals. According to the classification of the National Bureau of Statistics, the country consisted of six geographic regions (Supplementary Figure 1). First, from each region, we selected one teaching hospital as the regional coordinating center. Subsequently, three cities were randomly selected from that region. Then, from each selected city, we chose one tertiary hospital as a survey site based on our capacity and available resources. In total, 24 tertiary hospitals were included as survey sites (Supplementary Table 1).

The participating hospitals should meet the following criteria: 1) serum ferritin and hemoglobin were routinely tested at the local laboratory; 2) the laboratory facilities were examined by a quality control team comprising senior technicians, and 3) more than 400 pregnant women attending the hospital antenatal clinic during the survey period.

### Patient eligibility

Our clinical investigators consecutively enrolled eligible pregnant women, regardless of their gestational age, who were visiting the selected hospitals for antenatal care. Eligibility criteria were: completed an antenatal visit between 19 September and 20 November 2016; agreed to take a blood test and complete the questionnaire survey, by signing an informed consent form. Those pregnant women who had been participating in another clinical study during the same period were excluded.

### Outcome measures

The primary outcomes were the presence of anemia and IDA. According to the Chinese Guideline for Diagnosis and Treatment of IDA during Pregnancy [13], anemia was diagnosed by hemoglobin less than 110 g/l, and IDA was diagnosed by serum ferritin concentration less than 20 μg/l and hemoglobin less than 110 g/l.

All eligible participants were subjected to hemoglobin and serum ferritin examination during their antenatal visit. Blood samples were tested at the local laboratories using standard qualified methods.

We also documented previously clinician-diagnosed IDA during this pregnancy (i.e. first, second, or third trimester), which have been diagnosed by clinicians and recorded in medical charts.

### Data collection

We used two structured questionnaires which had been pilot tested to collect information (Supplementary Table 2 and Table 3). The first questionnaire consisted of items regarding participant demographics, history of pregnancy and childbirth, gestational comorbidities, behavior and lifestyle, previously clinician-diagnosed IDA, and history of other gestational comorbidities. The second clinician- reported questionnaire was completed by manually reviewing electronic medical records (EMRs), including anthropometrics (e.g. height and weight), physical examinations, laboratory results (e.g. hemoglobin, serum ferritin, red blood cell count and leucocyte count), treatment of IDA, and maternal complications during pregnancy.

A centralized online electronic data capturing system was set up to collect, review, and store data. Participants either completed the questionnaire through WeChat or webpages, or a hardcopy version which was subsequently uploaded to the central system by trained investigators. Logic verification rules and reference ranges were implemented for data verification.

From each participating woman, we thoroughly collected the following information: demographic characteristics (maternal age, ethnicity, education level, local resident status, registered residential place (urban or rural) and annual family income), gestational characteristics (gestational weeks of pregnancy, multiple gestations, parity, use of assisted reproductive technology (ART), and nausea or vomiting during the first trimester (NVP)), anthropometrics (height, pre-pregnancy weight and current weight), behavior and habits (e.g. active or passive smoking, meat intake), nutrients supplementation (intake of folic acid, multivitamins and calcium supplements), and gestational co-morbidities (e.g. hematological diseases, HBV infection, hypertension, gynecological diseases, diabetes (Type I or Type II), thyroid and autoimmune diseases). Gestational co-morbidities were defined as those conditions diagnosed before conception by clinicians, or diagnoses at the first antenatal visit.

### Sample size estimation

Prevalence data reported from a retrospective study was used for the sample size calculation [14]. Assuming a conservative prevalence estimate of maternal IDA being 3.96% (i.e. *p* ≈4%), the corresponding d (tolerance error) was taken to be 0.004 (one-tenth of *p* ), with *Z*_α/2_ =1.96 and 2-tailed α = 0.05, the sample size required was *n* = 9220.

Taking the available study resources and logistics into consideration, the investigators decided to increase the sample size to *n* = 12,000. Each regional coordinating center was supposed to enroll at least 800 pregnant women, and the other 18 hospitals were expected to consecutively recruit over 400 participants at each site.

### Statistical analysis

We summarized and compared baseline characteristics of pregnant women according to trimesters. Pearson’s chi-square or Fisher’s exact test were applied to categorical variables, and one-way ANOVA or Kruskal-Wallis test to continuous variables. Pre-pregnancy BMI was categorized into underweight (BMI < 18.5 kg/m^2^), normal (BMI ≥18.5 kg/m^2^ and < 25 kg/m^2^), overweight and obese (BMI ≥ 25 kg/m^2^). We included women with complete data in our analyses.

We compared the observed hemoglobin and serum ferritin levels by study subgroup (singletons; multiple gestations) and by trimesters (first; second; third trimester), using means or medians as appropriate. Similarly, the frequencies and prevalence of anemia and IDA were compared with respect to trimesters and subgroups of interest.

To determine the risk factors associated with anemia and IDA, we considered all plausible factors from the literature, namely, demographic characteristics, gestational characteristics, anthropometry indicators, behavior and habits, nutrients supplementation, and gestational co-morbidities; details of these variables were given in the data collection section. Initially, associations with potential factors were explored by fitting univariate logistic regression models accounting for trimesters, with the significance level set at *p* = 0.1. Next, we fitted mixed-effects logistic regression models for anemia and IDA separately. In view of the inherent correlation of observations due to the clustering effects of different hospitals, a mixed-effects logistic regression model with (24) random hospital effects was considered appropriate for our study setting.

### Sensitivity analysis

In our study, we developed two sensitivity analyses. Firstly, to compare the prevalence of anemia during pregnancy with dissimilar races and settings, we used the different hemoglobin concentration thresholds for anemia diagnosis from other guidelines, involving UK guideline (i.e., anemia was defined as hemoglobin concentration < 110 g/l in the first trimester, < 105 g/l in second and third trimesters and < 100 g/l postpartum and serum ferritin concentration < 30 μg/l) [15] and WHO guideline (i.e., anemia was diagnosed as hemoglobin concentration < 110 g/l in the first and third trimesters, and < 105 g/l in the second trimester) [16], and reported the corresponding prevalence. Secondly, we conducted a mixed-effects logistic regression model with random hospital effects by removing pregnant women with a history of hematological diseases before pregnancy.

## Results

Using a multi-stage sampling method, 24 hospitals from 21 cities of 16 provinces or municipal cities in China were enrolled in this study (Supplementary Figure 1). In total, 12,466 eligible pregnant women were invited to participate. After removing 63 refusals, 12,403 participants were included in the analysis (response rate: 99.5%). The number of enrollments varied from 800 to 906 among the regional coordination centers, whereas recruitments were lower in the other hospitals (range: 400 to 443, Supplementary Table 1). The flow chart of the included sample of participants was given in Supplementary Figure 2.

### Baseline characteristics

For our sample, 1018 women (8.2%) were at the first trimester, 3487 (28.1%) at the second, and 7898 (63.7%) at the third. Table 1 presents the baseline characteristics of the participants by trimesters. The mean maternal age was 30 (SD 4) years, with the majority of participants being of Han ethnicity (93.1%), living in urban area (51.5%), attained higher education level (78.8%), and had annual family income over 30 thousand yuan (90.6%) (Table 1). It is evident that pregnant women at the three trimesters differs in a number of baseline characteristics (*p* < 0.05).

**Table 1:** Characteristics of participants by trimesters

Characteristics	Total (n,%)	First trimester (n,%)	Second trimester (n,%)	Third trimester (n,%)	*P value*
Number	12,403 (100.0)	1018 (8.21)	3487 (28.11)	7898 (63.68)	____
Regions					
Southwest	2105 (16.97)	214 (21.02)	693 (19.87)	1198 (15.17)	
East	2008 (16.19)	123 (12.08)	484 (13.88)	1401 (17.74)	
Central-south	2044 (16.48)	181 (17.78)	601 (17.24)	1262 (15.98)	
Northeast	2179 (17.57)	38 (3.73)	469 (13.45)	1672 (21.17)	
Northwest	2051 (16.54)	231 (22.69)	711 (20.39)	1109 (14.04)	
North	2016 (16.25)	231 (22.69)	529 (15.17)	1256 (15.90)	< 0.001^1^
Maternal age					
< 35 years	10,595 (85.42)	893 (87.72)	2974 (85.29)	6728 (85.19)	
≥ 35 years	1808 (14.58)	125 (12.28)	513 (14.71)	1170 (14.81)	0.09^1^
Ethnicity					
Han	11,542 (93.06)	959 (94.20)	3257 (93.40)	7326 (92.76)	
Others	861 (6.94)	59 (5.80)	230 (6.60)	572 (7.24)	0.148^1^
Education level					
≥ 17 years	1376 (11.09)	120 (11.79)	372 (10.67)	884 (11.19)	
13–16 years	8403 (67.75)	699 (68.66)	2306 (66.13)	5398 (68.35)	
10–12 years	1698 (13.69)	140 (13.75)	522 (14.97)	1036 (13.12)	
≤ 9 years	926 (7.47)	59 (5.80)	287 (8.23)	580 (7.34)	0.02^1^
Local residents					
No	1394 (11.24)	120 (11.79)	463 (13.28)	811 (10.27)	
Yes	11,009 (88.76)	898 (88.21)	3024 (86.72)	7087 (89.73)	< 0.001^1^
Registered residential place					
Urban	6367 (51.33)	516 (50.69)	1806 (51.79)	4045 (51.22)	
Rural	6036 (48.67)	502 (49.31)	1681 (48.21)	3853 (48.78)	0.776^1^
Annual family income (thousand yuan)					
< 30	1167 (9.41)	72 (7.07)	320 (9.18)	775 (9.81)	
30–79.9	3427 (27.63)	263 (25.83)	994 (28.51)	2170 (27.48)	
80–119.9	3351 (27.02)	301 (29.57)	1010 (28.96)	2040 (25.83)	
120–199.9	2549 (20.55)	228 (22.40)	686 (19.67)	1635 (20.70)	
≥ 200	1909 (15.39)	154 (15.13)	477 (13.68)	1278 (16.18)	< 0.001^1^
Multiple gestations					
No	12,121 (97.84)	996 (96.70)	3372 (98.16)	7753 (97.73)	
Yes	282 (2.16)	22 (3.30)	115 (1.84)	145 (2.27)	< 0.001^1^
Parity					
Nulliparity	8268 (66.66)	713 (70.04)	2290 (65.67)	5265 (66.66)	
Multiparity	4135 (33.34)	305 (29.96)	1197 (34.33)	2633 (33.34)	0.034^1^
Use of ART					
No	11,921 (96.11)	969 (95.19)	3296 (94.52)	7656 (96.94)	
Yes	482 (3.89)	49 (4.81)	191 (5.48)	242 (3.06)	< 0.001^1^
NVP					
No	2957 (23.84)	167 (16.40)	715 (20.50)	2075 (26.27)	
Slight	7496 (60.44)	671 (65.91)	2196 (62.98)	4629 (58.61)	
Severe	1950 (15.72)	180 (17.68)	576 (16.52)	1194 (15.12)	< 0.001^1^
Pre-pregnancy BMI					
Underweight	2147 (17.31)	178 (17.49)	625 (17.92)	1344 (17.02)	
Normal weight	8925 (71.96)	738 (72.50)	2467 (70.75)	5720 (72.42)	
Overweight and obese	1331 (10.73)	102 (10.02)	395 (11.33)	834 (10.56)	0.405^1^
Active or passive smoking					
No	7710 (57.37)	584 (62.46)	2178 (62.65)	4948 (62.16)	
Yes	4693 (42.63)	434 (37.54)	1309 (37.35)	2950 (37.84)	0.004^1^
Meat intake (kg/week)^a^	0.30 (0.15–0.45)	0.15 (0.10–0.35)	0.25 (0.10–0.4)	0.30 (0.15–0.50)	< 0.001^2^
Intake of folic acid					
No	2077 (16.75)	181 (17.78)	530 (15.20)	1366 (17.30)	
Yes	10,326 (83.25)	837 (82.22)	2957 (84.80)	6532 (82.70)	0.014^1^
Intake of multivitamins					
No	4305 (34.71)	517 (50.79)	1322 (37.91)	2466 (31.22)	
Yes	8098 (65.29)	501 (49.21)	2165 (62.09)	5432 (68.78)	< 0.001^1^
Intake of calcium					
No	4367 (35.21)	887 (87.13)	1664 (47.72)	1816 (22.99)	
Yes	8036 (64.79)	131 (12.87)	1823 (52.28)	6082 (77.01)	< 0.001^1^
Hematological diseases					
No	11,782 (94.99)	987 (96.95)	3365 (96.50)	7430 (94.07)	
Yes	621 (5.01)	31 (3.05)	122 (3.50)	468 (5.93)	< 0.001^1^
HBV infection					
No	12,097 (97.53)	999 (98.13)	3405 (97.65)	7693 (97.40)	
Yes	306 (2.47)	19 (1.87)	82 (2.35)	205 (2.60)	0.323^1^
Hypertension					
No	12,363 (99.68)	1012 (99.41)	3473 (99.60)	7878 (99.75)	
Yes	40 (0.32)	6 (0.59)	14 (0.40)	20 (0.25)	0.128^1^
Gynecological diseases					
No	11,719 (94.49)	933 (91.65)	3288 (94.29)	7498 (94.94)	
Yes	684 (5.51)	85 (8.35)	199 (5.71)	400 (5.06)	< 0.001^1^
Diabetes					
No	12,346 (99.51)	1013 (99.51)	3470 (99.51)	7863 (99.56)	
Yes	57 (0.49)	5 (0.49)	17 (0.49)	35 (0.44)	0.938^1^
Thyroid diseases					
No	11,734 (94.61)	977 (95.97)	3295 (94.49)	7462 (94.48)	
Yes	669 (5.39)	41 (4.03)	192 (5.51)	436 (5.52)	0.131^1^
Autoimmune diseases					
No	12,356 (99.62)	1010 (99.21)	3461 (99.25)	7885 (99.84)	
Yes	47 (0.38)	8 (0.79)	26 (0.75)	13 (0.16)	< 0.001^1^
Digestive system diseases					
No	11,881 (95.79)	949 (93.22)	3331 (95.53)	7601 (96.24)	
Yes	522 (4.21)	69 (6.78)	156 (4.47)	297 (3.76)	< 0.001^1^

^1^From Pearson’s chi-square test

^2^From Kruskal-Wallis rank test

^a^Median (lower quartile-upper quartile)

*ART* assisted reproductive technology; *NVP* nausea or vomiting during pregnancy; *HBV* hepatitis B viral; *BMI* Body Mass Index

### Hemoglobin and serum ferritin

Table 2 summarizes the hemoglobin and serum ferritin levels by study subgroups and trimesters. It appears that both hemoglobin and serum ferritin tend to decrease with advancing trimesters, especially among women with multiple gestations (Table 2).

**Table 2:** Average hemoglobin and serum ferritin levels of participants by trimesters

Indicator	Sample	Total	First trimester	Second trimester	Third trimester	*P value*
Hemoglobin: mean (SD)	All pregnancies	118.38 (11.52)	126.78 (10.20)	118.93 (11.27)	117.06 (11.32)	< 0.001^1^
	Singletons	118.43 (11.49)	126.86 (10.23)	118.99 (11.25)	117.10 (11.26)	< 0.001^1^
	Multiple gestations	116.47 (12.88)	123.44 (7.91)	117.17 (11.73)	114.86 (13.98)	0.01^1^
Serum ferritin: median (lower quartile-upper quartile)	All pregnancies	20.60 (11.79–36.97)	54.39 (34.5–94.01)	28.60 (16.40–50.50)	16.70 (10.20–27.00)	< 0.001^2^
	Singletons	20.57 (11.7–36.80)	54.20 (34.37–93.35)	28.74 (16.40–50.49)	16.63 (10.20–26.9)	< 0.001^2^
	Multiple gestations	23.65 (13.9–44.44)	75.7 (35.30–125.6)	25.0 (13.9–55.25)	19.7(12.1–35.00)	< 0.001^2^

^1^From One-way ANOVA

^2^From Kruskal-Wallis rank test

### Prevalence of anemia and IDA

Table 3 presents the frequencies and prevalence of anemia and IDA by study subgroups and trimesters. A total of 2460 women were diagnosed with anemia; of whom 50 were identified at the first trimester, 577 at the second, and 1833 at the third. The overall prevalence of anemia was 19.8% (2460/12403), and 4.9% (50/1018) in the first trimester, 16.6% (577/3487) in the second trimester and 23.2% (1833/7898) in the third trimester. Besides, 13.9% (1720/12403) pregnant women were diagnosed with IDA, corresponding to 2.0% (20/1018) at the first trimester, 8.4% (293/3487) at the second, and 17.8% (1407/7898) at the third (Table 3).

**Table 3:** Frequencies and prevalence of anemia and IDA

Outcomes	Sample	Total (n, %)	First trimester (n,%)	Second trimester (n,%)	Third trimester (n,%)	*P value*^1^
**Anemia**	All pregnancies	2460 (19.84)	50 (4.91)	577 (16.55)	1833 (23.21)	< 0.001
	Singletons	2379 (19.63)	49 (4.92)	547 (16.23)	1783 (23.00)	< 0.001
	Multiple gestations	81 (28.72)	1 (4.55)	30 (26.09)	50 (34.48)	0.011
	Maternal age < 35	2055 (19.40)	42 (4.70)	477 (16.04)	1536 (22.83)	< 0.001
	Maternal age ≥ 35	405 (22.40)	8 (6.40)	100 (19.49)	297 (25.38)	< 0.001
	Nullipara	1520 (18.39)	31 (4.36)	372 (16.25)	1117 (21.22)	< 0.001
	Multipara	940 (22.73)	19 (6.23)	205 (17.13)	716 (27.19)	< 0.001
**IDA**	All pregnancies	1720 (13.87)	20 (1.96)	293 (8.41)	1407 (17.82)	< 0.001
	Singletons	1662 (13.72)	19 (1.91)	276 (8.19)	1367 (17.63)	< 0.001
	Multiple gestations	58 (20.57)	1 (4.55)	17 (14.78)	40 (27.59)	0.006
	Maternal age < 35	1449 (13.68)	17 (1.90)	247 (8.31)	1185 (17.62)	< 0.001
	Maternal age ≥ 35	271 (14.99)	3 (2.40)	46 (8.97)	222 (18.97)	< 0.001
	Nullipara	1031 (12.47)	10 (1.40)	174 (7.40)	847 (16.09)	< 0.001
	Multipara	689 (16.66)	10 (3.28)	119 (9.94)	560 (21.27)	< 0.001
**Previously clinician-diagnosed IDA**	All pregnancies	3796 (30.61)	53 (5.21)	594 (17.03)	3149 (39.87)	< 0.001
	Singletons	3699 (30.52)	52 (5.22)	564 (16.73)	3083 (39.77)	< 0.001
	Multiple gestations	97 (34.52)	1 (4.55)	30 (26.09)	66 (45.52)	< 0.001
	Maternal age < 35	3226 (30.45)	48 (5.38)	510 (17.15)	2668 (39.66)	< 0.001
	Maternal age ≥ 35	570 (31.53)	5 (4.00)	84 (16.37)	481 (41.11)	< 0.001
	Nullipara	2415 (29.21)	37 (5.19)	360 (15.72)	2081 (38.33)	< 0.001
	Multipara	1381 (33.40)	16 (5.25)	234 (19.55)	1131 (42.95)	< 0.001

^1^From Pearson’s chi-square test

*IDA* iron deficiency anemia

**Fig. 1:**
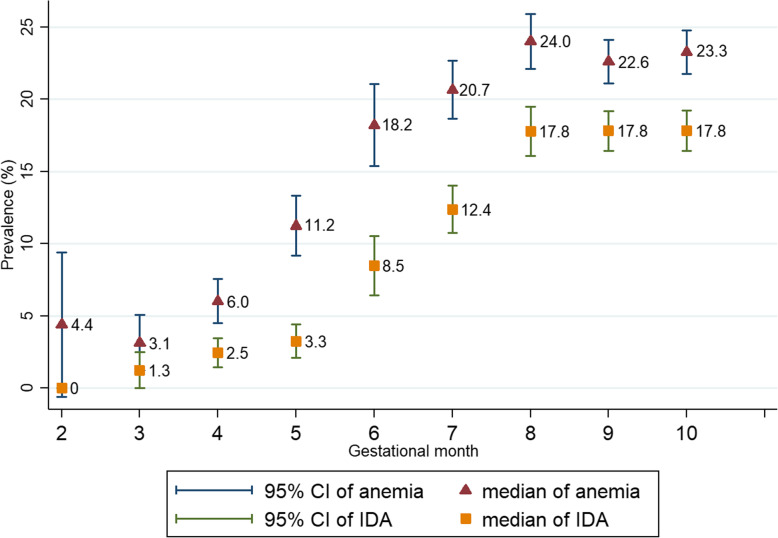
Prevalence of anemia and IDA at each gestational month

Compared with singletons, women with multiple gestations experienced higher prevalence of anemia (28.7% vs. 19.6%) and IDA (20.6% vs. 13.7%). Similarly, pregnant women with more than 35 years (22.4% vs. 19.4% for anemia, 15.0% vs. 13.7% for IDA) and multiparity (22.7% vs. 18.4% for anemia, 16.7% vs. 12.5% for IDA) presented higher prevalence of anemia and IDA than the counterparts (Table 3). The prevalence of anemia and IDA increased with trimesters, and peaked at the eighth month (24.0% for anemia and 17.8% for IDA) (Fig. 1).

In addition, 5.2% (53/1018), 17.0% (594/3487) and 39.9% (3149/7898) pregnancies were previously clinician-diagnosed with IDA at the three trimesters, respectively (Table 3). The prevalence also increased with the gestational month (Supplementary Figure 3).

**Fig. 2:**
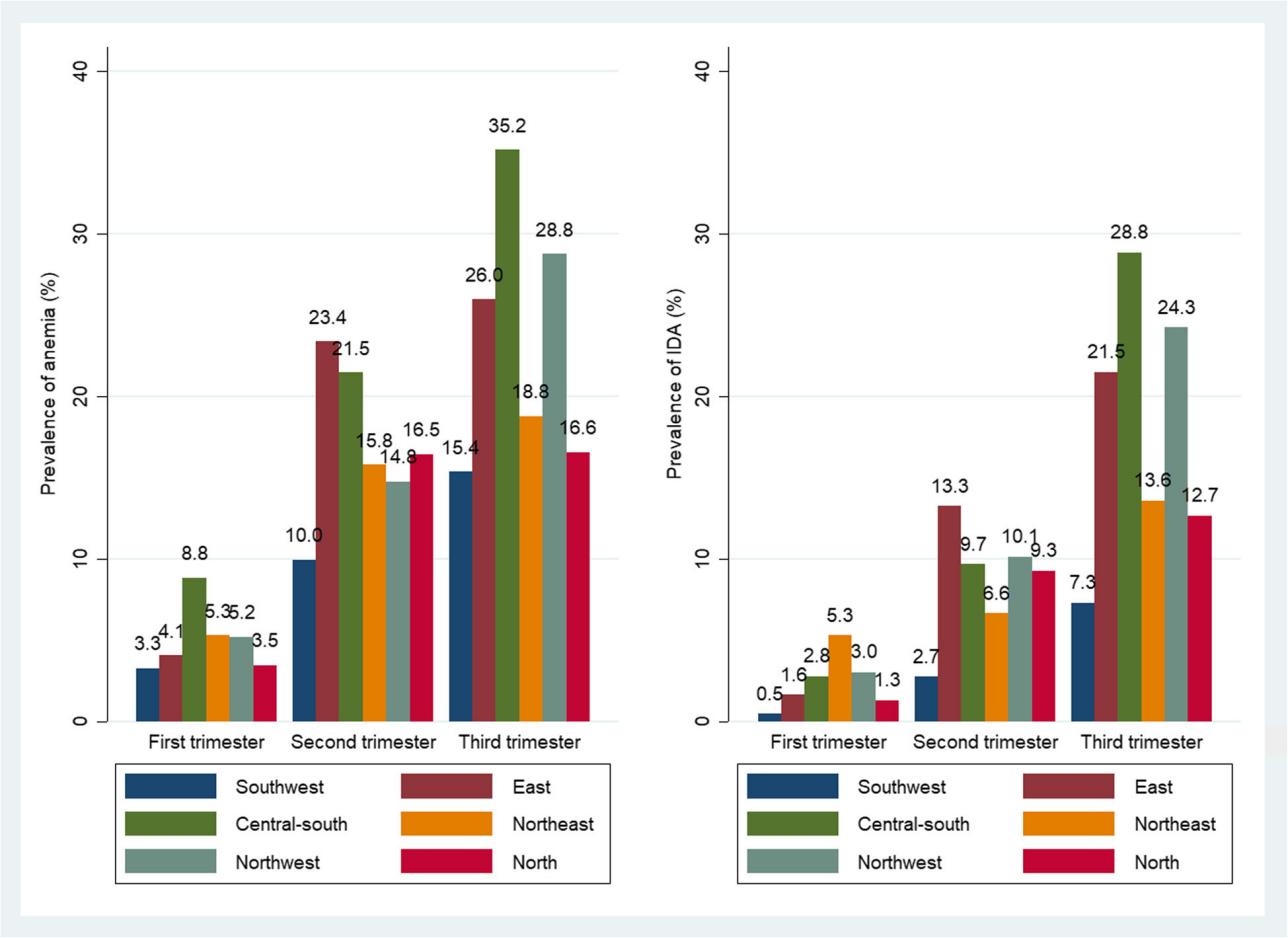
Prevalence of anemia and IDA among the six regions of China

Figure 2 shows that the prevalence of anemia and IDA varies substantially among the six regions of China. The southwest reported the lowest prevalence of anemia (10.0%) and IDA (15.4%), whereas the east area had the highest prevalence in the second trimester (anemia 23.4% and IDA 13.3%), and central-south had the highest prevalence in the third trimester (anemia 35.2% and IDA 28.8%) (Fig. 2).

### Risk factors

As shown in Table 4, the mixed-effects logistic regression analysis identified the following factors independently associated with a higher risk of anemia: late trimesters, maternal age 35 years or older, non-local residents, multiple gestations, multiparity, pre-pregnancy underweight, severe NVP during early pregnancy, pre-existing IDA and other hematologic diseases. On the other hand, women who consume multivitamins and more meat were associated with a lower risk of anemia. (Table 4). Similar influencing factors in the same direction of associations were found for IDA, with the exception of maternal age (not significant; Table 4).

**Table 4:** Risk factors for anemia and IDA

Factors	Anemia: adjusted OR (95% CI)^a^	IDA: adjusted OR (95% CI)^b^
Trimester		
Second vs. First	4.11 (3.02–5.57)	4.99 (3.13–7.95)
Third vs. First	6.10 (4.52–8.22)	11.72 (7.43–18.49)
Maternal age (≥35 vs. < 35 years)	1.22 (1.06–1.40)	–
Local residents (No vs. Yes)	1.18 (1.02–1.36)	1.20 (1.02–1.42)
Multiple gestations (Yes vs. No)	1.54 (1.13–2.09)	1.90 (1.38–2.62)
Parity (Multiparity vs. Nulliparity)	1.18 (1.06–1.31)	1.28 (1.14–1.44)
Pre-pregnancy BMI		
Underweight vs. Normal weight	1.32 (1.17–1.49)	1.30 (1.13–1.49)
Overweight and obese vs. Normal weight	0.67 (0.57–0.80)	0.62 (0.51–0.77)
NVP		
Slight vs. No	1.05 (0.93–1.18)	1.06 (0.93–1.22)
Severe vs. No	1.21 (1.04–1.41)	1.21 (1.02–1.44)
Intake of multivitamins (Yes vs. No)	0.58 (0.52–0.65)	0.49 (0.43–0.55)
IDA before pregnancy (Yes vs. No)	2.92 (2.41–3.54)	2.81 (2.28–3.48)
Other hematologic diseases (Yes vs. No)	8.25 (5.19–13.13)	2.00 (1.25–3.20)
Meat intake (kg/week)	0.81 (0.68–0.96)	0.80 (0.65–0.98)

^a^Logistic mixed regression model adjusting for education level, registered residential place, annual family income, use of ART, and intake of folic acid

^b^Logistic mixed regression model adjusting for education level, annual family income, intake of folic acid, gynecological diseases, and thyroid diseases

*OR* odds ratio; *CI* confidence interval; *ART* assisted reproductive technology; *NVP* nausea or vomiting during pregnancy; *BMI* Body Mass Index

### Sensitivity analysis

By using the different thresholds referring to anemia diagnosis, we found the overall prevalence of anemia by laboratory tests was 6.43% (796/12403) in UK guideline, corresponding to 4.91% (50/1018) in the first trimester, 7.66% (267/3487) in the second, and 6.09% (481/7898) in the third. Combined with the thresholds of serum ferritin concentration less than 30 μg/l, the prevalence of overall IDA was 5.14% (637/12403), corresponding to 2.26% (23/1018) in the first, 5.51% (192/3487) in the second and 5.34% (422/7898) in the third trimester. Besides, compared with WHO’s recommendation, we found the overall prevalence of anemia was 17.33% (2150/12403), corresponding to 4.91% (50/1018) in the first, 7.66% (267/3487) in the second, and 23.21% (1833/7898) in the third). In addition, the sensitivity analysis by removing pregnant women with hematological diseases diagnosed before pregnancy confirmed the similar results (not presented for brevity).

## Discussion

### Prevalence of anemia and IDA

Using a large representative sample of pregnant women, we found that 19.8% of Chinese women had anemia and 13.9% had IDA during pregnancy, and the conditions became more prevalent over the progression of gestation. The prevalence of anemia and IDA also varied substantially across geographic regions of China, from the lowest in the southwest to the highest in the central-south.

In China, anemia in pregnancy was diagnosed as hemoglobin concentrations < 110 g/l throughout three trimesters. When we used the newest UK’s guideline for assessing anemia, we found the prevalence of anemia was consistent in the first trimester, but much lower than those diagnosed by Chinese guideline for overall anemia (i.e., 6.43% vs. 19.84%). On the other hand, WHO’s recommendation defined the different threshold in the second trimester only, thus having slightly lower overall prevalence of anemia (i.e., 17.33% vs. 19.84%). Due to physiological expansion of plasma volume in pregnancy, especially in the second and third trimesters [17], setting different thresholds for defining anemia diagnosis in pregnancy seems to be plausible. However, the current hemoglobin concentrations thresholds during pregnancy derived from non-pregnant populations, and there was limited evidence to clarify the association between those thresholds and subsequently clinical outcomes [18]. Additionally, it was reported that racial disparities were not negligible in maternal hemoglobin concentrations and pregnancy outcomes [19]. Therefore, we adopted the Chinese guideline in our main analysis, being consistent with Chinese practice, and supplemented a sensitivity analysis to compare the prevalence of anemia according to other thresholds.

On the other hand, IDA was defined as anemia plus serum ferritin < 20 μg/l in our study, whereas different thresholds were used in other countries [20, 21], for example, < 10 μg/l USA [22]; < 15 μg/l Korea [23]; < 12–15 μg/l Europe [5]; and < 30 μg/l Switzerland [24] and UK [15]. Accordingly, the direct comparison of prevalence of IDA among countries were actually difficult. In sensitivity analysis, we defined IDA according to UK’s guideline, thus presenting the lower prevalence of overall IDA (5.14% vs. 13.87%). In addition, our study found rapid decline in hemoglobin and serum ferritin levels with advancing trimesters, consistent with the literatures [5, 25, 26]. The prevalence of anemia and IDA peaked at the eighth gestational month according to diagnosis in Chinese guideline.

A recent systematic analysis (2011) reported that the global prevalence of anemia among pregnant women was 38% [3]. In developed countries, the prevalence was lower (for example, anemia: 12.8% in Canada [27], 15.8% in France [28] and 8.8% in U. S [29]; IDA: 16% in Belgian, and 3% in Switzerland), but the prevalence remained high in developing nations (for example, anemia: 53.4% in Congo [7]); IDA: 51.3% in Egypt [6] and 44.5% in Saudi Arabia [30]). Besides, the prevalence of anemia in pregnant population varied across Asia-pacific regions, such as 25.7% in Korea [5], 20–25% in Australia [31–33] and 50% in India [34]. According to the estimates by WHO, the lowest prevalence of anemia during pregnancy occurred in Western Pacific region (24.3%) and the highest prevalence in South-East Asia region (48.7%) [35].

Our findings were similar to those from developed countries. This suggests that pregnancy care for the Chinese population has improved over the past decades. However, due to different diagnostic criteria adopted by studies, one should interpret the findings with caution [36–38].

In addition, we found the prevalence of anemia and IDA varied substantially among the six regions of China. Except for the impact of socioeconomic status, the difference of dietary habit and region-epidemic of thalassemia among Chinese provinces and races, might play important role in diverse distribution of anemia and IDA. These factors were also used to illustrate the high prevalence of anemia in South-East Asia, such as high epidemic of thalassemia in Thailand, Laos and Philippines, and low-iron dietary intake and sickle-cell anemia in India [39–41].

### Risk factors for anemia and IDA

Several risk factors were identified for anemia and IDA. The magnitudes of association for pertinent factors such as advanced trimesters, multiple gestations, IDA prior to pregnancy, and hematological diseases other than IDA, were relatively strong (adjusted odds ratio over 2). The results were consistent with studies on non-Chinese populations [42–45], and have important implications for managing pregnant women with these characteristics who may be at elevated risk. The sensitivity analysis by excluding those with previously diagnosed IDA or anemia have further confirmed the apparent adverse effect of these factors on the development of anemia or IDA.

In addition, several other factors were found to be moderately associated with the prevalence of anemia and IDA (adjusted odds ratios between 1.2 and 1.3), including non-local residents, multiparity, severe NVP, and pre-pregnancy underweight, similar to findings reported in the literature [23, 45–50]. These factors should also be taken into account when planning for interventions to improve the anemia and IDA status of Chinese pregnant women.

### Supplementation and screening

Multivitamins supplement and meat intake were associated with lower risk of anemia and IDA. Multivitamins products, which contain vitamins and minerals including iron [51, 52], are commonly consumed in China. The finding concerning meat intake was consistent with previous studies in Ethiopia [24, 53, 54], in which IDA was found more prevalent in regions with iron-poor diets such as central south, northwest, and east China [23, 55].

Prophylactic iron supplementation for pregnant women is a controversial issue [36–38]. Although the current Chinese guideline does not address such supplementation [13], routine iron supplementation during pregnancy have been suggested in 24 hospitals. Accordingly, the prevalence of anemia and IDA were substantially lower than national investigation conducted before 20 years in China [12]. Our findings underlined that iron supplements and dietary improvement may be warranted for susceptible and high-risk population subgroups, such as pregnant women with multiple gestations, more than 35 years and multiparity. It has been recommended that all pregnant women should be screened for anemia during pregnancy [22, 56, 57]. However, the timing of screening remains uncertain. At present, the Chinese guideline recommends screening of hemoglobin at the first antenatal visit, with repeated screening every 8–10 weeks [13]. Our result supports such screening, especially at the advanced trimesters. In addition, although serum ferritin test may be impacted by inflammatory or infectious conditions, a routine marker for diagnosis of iron deficiency was advisable during pregnancy in light of reliably automated operation and obvious cost-effectiveness compared with other markers (e.g., transferrin receptor) [20, 58]. A concurrent test for inflammatory markers (e.g., C-reactive protein) is considered to exclude other reactive causes [20, 59].

### Strengths and limitations

Our study has several advantages. In this nationwide survey, a multi-stage sampling method was applied to select the data collection sites. To our knowledge, this was the largest study ever undertaken in China to investigate the prevalence and risk factors of anemia and IDA during pregnancy. Moreover, rigorous methods were adopted for data collection, ensuring high quality information obtained across the country with minimal missing values. The large sample size has enabled the estimation of overall prevalence of anemia and IDA, as well as facilitated the comparison of rates among regions, population subgroups, and across trimesters.

Several limitations should be taken into consideration. Firstly, due to logistics problem, hemoglobin and serum ferritin were tested at local laboratories, where the diagnostic accuracy may vary slightly. To circumvent the problem, each participating tertiary hospital was required to be equipped with qualified facilities and professional technicians, and a laboratory quality control team was set up to minimize measurement errors, thus ensuring the reliability and comparability of results. Secondly, random sampling for hospital selection was not feasible due to the vastness of regions and economic constraints. We chose this approach to optimize the balance between available resource and the representativeness of our study locations. Thirdly, all the participating data collection sites were tertiary hospitals with laboratory facilities, the generalizability of findings may be slightly compromised without the inclusion of smaller hospitals and community clinics.

## Conclusion

The prevalence of anemia and IDA during pregnancy are similar to those from developed countries and vary across regions in China. However, due to different diagnostic criteria adopted, one should compare the findings with other studies with caution. Non-local residents, multiple gestations, multiparity, pre-pregnancy underweight, severe NVP, hematologic diseases before pregnancy and other hematologic diseases rather than IDA were associated with higher risks of anemia and IDA. Our study highlights the need for developing and implementing a rigorous management plan to control pregnancy anemia and IDA in China.

## Supplementary Information

**Additional file 1 Figure S1** The distribution of six regions and selected cities among China. **Figure S2** The flow chart of included population. **Figure S3** Median and 95% confidence intervals for prevalence of previously diagnosed IDA categorized by gestational month. **Table S1** List of 24 selected hospitals in our study and the number of included pregnant women. **Table S2** Survey on iron deficiency among pregnant women in China (Pregnant Women Investigation). **Table S3** Survey on iron deficiency among pregnant women in China (Doctor Investigation)

## Data Availability

The datasets analyzed during the current study are not currently available due to patient confidentiality reasons as well as the use of the same data for developing other manuscripts.

## Abbreviations

IDA: Iron deficiency anemia
EMR: Electronic medical records
ART: Assisted reproductive technology
NVP: Nausea or vomiting during pregnancy
HBV: Hepatitis B viral
BMI: Body mass index
aOR: Adjusted Odds Ratio
CI: Confidence interval
