# Supplementary material for: Prevalence of anemia and iron deficiency anemia in Chinese pregnant women (IRON WOMEN): a national cross-sectional survey
Source: BMC Pregnancy Childbirth. 2020 Nov 7;20:670. doi: 10.1186/s12884-020-03359-z (PMC7648278; doi:10.1186/s12884-020-03359-z)
Supplement: Supplementary file 1 — Additional file 1 Figure S1 The distribution of six regions and selected cities among China. Figure S2 The flow chart of included population. Figure S3 Median and 95% confidence intervals for prevalence of previously diagnosed IDA categorized by gestational month. Table S1 List of 24 selected hospitals in our study and the number of included pregnant women. Table S2 Survey on iron deficiency among pregnant women in China (Pregnant Women Investigation). Table S3 Survey on iron deficiency among pregnant women in China (Doctor Investigation) [file 12884_2020_3359_MOESM1_ESM.docx]

**Supplementary**


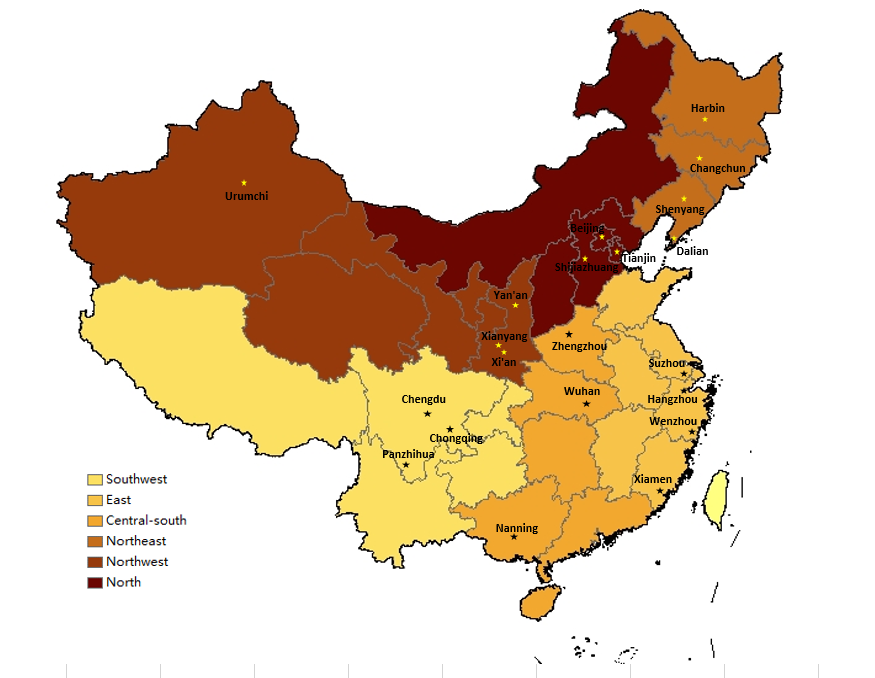


**Figure 1** The distribution of six regions and selected cities among China (Online Supplemental Material)

Of 12,466 eligible pregnant women receiving antenatal visits from September 19, 2016 to November 20, 2016 were screened

Of 12,403 eligible pregnant women were consecutively enrolled

Excluded 63 pregnant women refusing enrollment

Of one pregnant woman with missing of hemoglobin, and three pregnant women with missing of serum ferritin

Finally, 12402 and 12400 eligible pregnant women were used to examine the outcomes of  anemia and IDA in analysis, respectively

**Figure 2** The flow chart of included population (Online Supplemental Material)


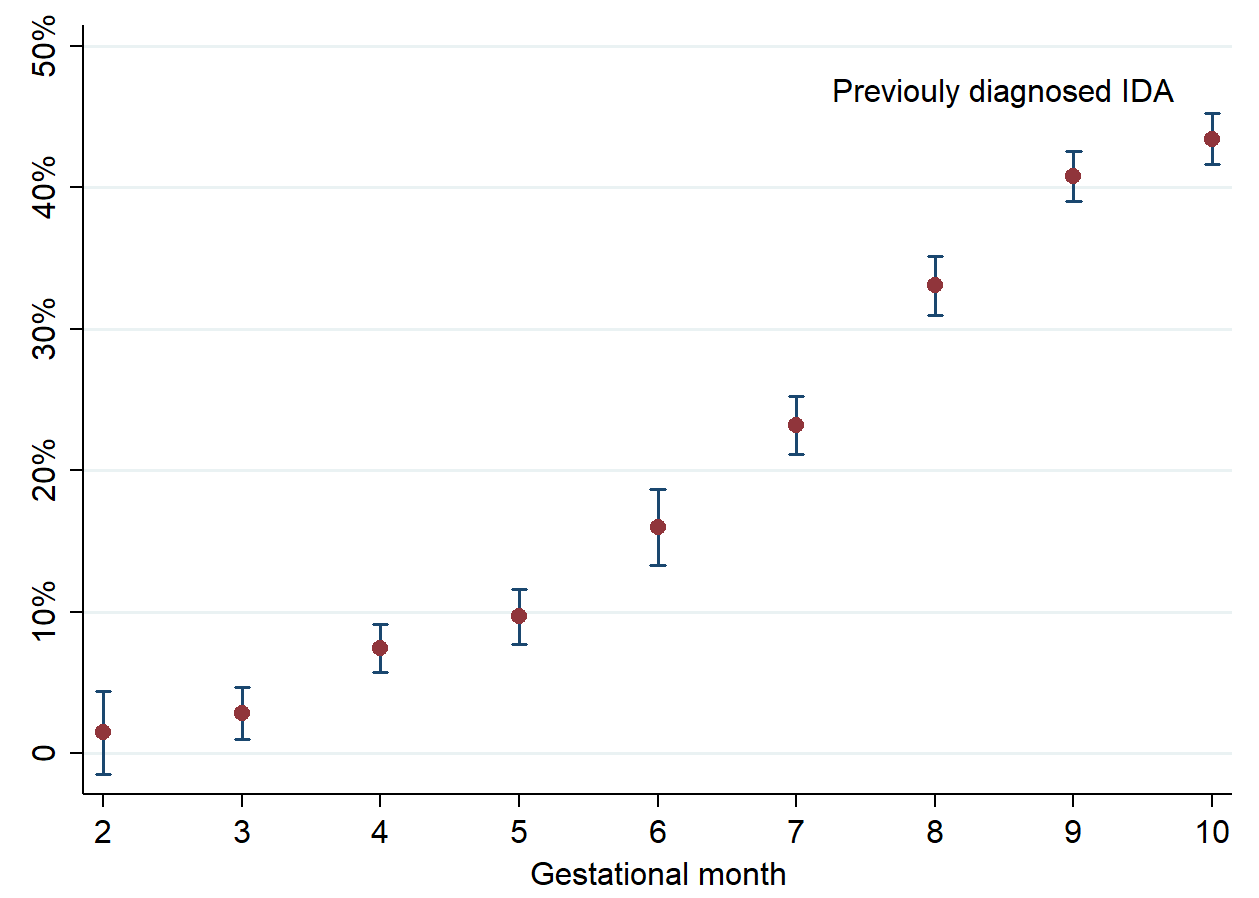


**Figure 3** Median and 95% confidence intervals for prevalence of previously diagnosed IDA categorized by gestational month (Online Supplemental Material)

**Table 1** List of 24 selected hospitals in our study and the number of included pregnant women (Online Supplemental Material)

| **Regions** | **Cities** | **Selected hospitals** | **Number of included individuals** |
| --- | --- | --- | --- |
| Southwest | Chengdu | West China Second University Hospital, Sichuan University^*^ | 861 |
|  | Chengdu | Sichuan Provincial Hospital for Women and Children | 400 |
|  | Panzhihua | Panzhihua Central Hospital | 404 |
|  | Chongqing | Chongqing Health Center for Women and Children | 440 |
| East | Hangzhou | Women’s Hospital School of Medicine, Zhejiang University^*^ | 803 |
|  | Xiamen | Xiamen Medical Center for Women and Children | 401 |
|  | Wenzhou | Wenzhou People's Hospital | 401 |
|  | Suzhou | Suzhou Second People's Hospital/ Suzhou Municipal Hospital | 403 |
| Central South | Wuhan | Union Hospital, Tongji Medical College, Huazhong University of Science and Technology ^*^ | 813 |
|  | Wuhan | Maternal and Child Health Hospital of Hubei Province | 414 |
|  | Zhengzhou | The First Affiliated Hospital of Zhengzhou University | 409 |
|  | Nanning | The First Affiliated Hospital of Guangxi Medical University | 825 |
| Northeast | Shenyang | Shengjing Hospital of China Medical University^*^ | 906 |
|  | Dalian | Maternal and Child Health Hospital of Dalian | 430 |
|  | Changchun | Changchun Obstetrics-Gynecology Hospital | 443 |
|  | Harbin | The Fourth Affiliated Hospital of Harbin Medical University | 400 |
| Northwest | Xi’an | Shanxi Provincial Hospital for Women and Children/ Northwest Women’s and Children’s Hospital ^*^ | 825 |
|  | Xianyang | The Second Affiliated Hospital of Shanxi University of Traditional Chinese Medicine | 402 |
|  | Yan'an | Yan'an University Affiliated Hospital | 424 |
|  | Urumchi | Maternal and Child Health Hospital of Urumchi, Xinjiang | 400 |
| North | Beijing | Beijing Obstetrics and Gynecology Hospital, Capital Medical University ^*^ | 810 |
|  | Tianjin | Tianjin Central Hospital of Gynecology Obstetrics | 400 |
|  | Shijiazhuang | The Fourth Hospital of Shijiazhuang/ Shijiazhuang Obstetrics and Gynecology Hospital | 405 |
|  | Beijing | Beijing Friendship Hospital, Capital Medical University | 401 |

* coordinator of each region.

**Table 2 Survey on iron deficiency among pregnant women in China**

**(Pregnant Women Investigation)**

**Survey Number: □□ Region Code □□ Hospital Code □□□□ Person Code**

**Instructions**

In order to understand your health status during pregnancy, and better protect the health of you and your newborn, we will investigate your pregnancy-related conditions and some personal information. We declare that all the information you provided will only be used for research purposes. Please tick or fill in the relevant information according to the actual situation, and all questions are single choice by default except for special instructions.

| **A Basic demographic characteristic** | | |
| --- | --- | --- |
| A1 | Maternal name | ________ |
| A2 | Maternal age | □□ Years |
| A3 | Telephone number | □□□□□□□□□□□ |
| A4 | Visit card number | ________ |
| A5 | Last menstrual period (LMP) | □□□□Year □□Month □□Day □0=Unclear |
| A6 | Ethnicity | □0 = Han □1= Others, specified____________________ |
| A7 | Education level | □□ Years |
| A8 | Occupation | □1 = Agriculture, forestry, animal husbandry, fishery, e.g. farmers, hunters, herdsmen, fishermen, etc.  □2 = Production, transportation, equipment operators, e.g. drivers, operators, welders, etc.  □3 = Service, e.g. cooks, waiters, barbers, salesmen, etc.  □4 = Office staff, e.g. secretary, bank clerk, clerk, etc.  □5 = Professional and technical personnel, e.g. medical staff, teachers, lawyers, architects, IT, editors, athletes, etc.  □6 = Management personnel, e.g. government officials, factory directors, managers, administrative cadres, etc.  □7 = None  □8 = Others |
| A9 | Marriage | □1= Married □2= Single □3= Widowed □4= Divorced |
| A10 | Husband age | □□ Years |
| A11 | Local resident (living more than 6 months) | □0 = No □1 = Yes |
| A12 | Registered residential place | □0= Urban □1= Rural |
| A13 | Number of family members | _____Person |
| A14 | Annual family income | □1= <30,000 yuan □2= 30,000-79,999 yuan  □3=80,000-11,999 yuan □4=12,000-19,999 yuan  □5=20,000-29,999 yuan □6= >30,000 yuan |
| **B Physical measurement** | | |
| B1 | Height | □□□cm |
| B2 | Pre-pregnancy weight | □□□kg |
| B3 | Current weight | □□□kg |
| **C Dietary habits** | | |
| C1 | Vegetarian | □0=No □1= Yes |
| C2 | Living habits since pregnancy (multiple choices) | □0= None □1= Smoke( >1 cigarette per day on average)  □2= Drink ( >50 ml per day on average) □3= Drugs |
| C3 | Milk consumption per day since pregnancy | □1= None □2= <100ml □3= 100-299ml □4=300-499 ml □5= ≥500ml |
| C4 | Red meat consumption since pregnancy (e.g. pork, beef, mutton, etc.) | _____times per week, ____liang per time |
| C5 | Egg consumption since pregnancy | _____times per week, ____gram per time |
| C6 | Do your family members, friends, or colleagues who live together smoke? | □0=No □1= Yes |
| **D Gestational characteristics** | | |
| D1 | Last pregnancy history | □1= First pregnancy this time □2 = less than 6 months  □3=6 months to 1 year (not including 1 year)  □4= 1-2 years (not including 2 years) □5= 2-3 years (not including 3 years) □6= 3-4 years (not including 4 years) □7= more than 4 years |
| D2 | Do you have any nausea or vomiting during pregnancy? | □0= None  □1= Mild (symptomatic, but does not affect daily life or mood)  □2=Severe (symptomatic, and have affected daily life or mood) |
| D3 | Do you have any the following symptom during last month? (multiple choices) | □0= None □1= Pale face □2= Weak □3= Palpitation  □4= Dizziness □5= Dyspnoea □6=Fatigue □7= Irritable  □8= irascible □9=Decreased attention □10= Hair loss |
| **E Supplementation of iron, folic acid, vitamins, and calcium** | | |
| E1 | Have you been diagnosed with iron deficiency anaemia (IDA) by clinicians since this pregnancy? | □0=None □1= Diagnosed during first trimester (gestational 1-3 months)  □2= Diagnosed during second trimester (gestational 4-7 months)  □3= Diagnosed during third trimester (gestational 8-10 months) |
| E2 | Do you have taken iron supplementation since pregnancy? | □0= None □1=LI-FEI-NENG □2= SU-LI-FEI □3= SHENG-XUE-NIGN  □4= Others, specified ________________ |
| E3 | Are you taking iron supplementations at present? | □0= None □1= LI-FEI-NENG □2= SU-LI-FEI  □3= SHENG-XUE-NING □4= Others, specified ________________ |
| E4 | How long have you taken iron supplementations? | □1= < 1 month □2= 1-3 months (not including 3 months)  □3= 3-6 months (not including 6 months) □4= ≥ 6 months |
| E5 | Do you take folic acid? | □0= None, then skip to **E9** □1= Yes |
| E6 | Starting timing for taking folic acid | □1= Pre-pregnancy □2= Gestational 1-3 months  □3= Gestational 4-7 months □4= Gestational 8-10 months |
| E7 | Duration of taking folic acid | □1= less than 1 month □2= 1-3 months (not including 3 months)  □3= 3-6 months (not including 6 months) □4= ≥ 6 months |
| E8 | Dose for taking folic acid | ______pieces per day; ______days per week |
| E9 | Do you take multi-vitamins? | □0= None, then skip to **E13** □1= Elevit  □2= Materna □3= Others, specified ________________ |
| E10 | Starting timing for taking multi-vitamins | □1= Pre-pregnancy □2= Gestational 1-3 months  □3= Gestational 4-7 months □4= Gestational 8-10 months |
| E11 | Duration of taking multi-vitamins | □1= less than 1 month □2= 1-3 months (not including 3 months)  □3= 3-6 months (not including 6 months) □4= ≥ 6 months |
| E12 | Dose for taking multi-vitamins | ______pieces per day; ______days per week |
| E13 | Do you take calcium? | □0= None, skip to **E17** □1= Caltrate  □2=DI-QIAO □3= Others, specified ________________ |
| E14 | Starting timing for taking calcium | □1= Pre-pregnancy □2= Gestational 1-3 months  □3= Gestational 4-7 months □4= Gestational 8-10 months |
| E15 | Duration of taking calcium | □1= less than 1 month □2= 1-3 months (not including 3 months)  □3= 3-6 months (not including 6 months) □4= ≥ 6 months |
| E16 | Dose for taking calcium | ______pieces per day; ______days per week |
| E17 | Have you taken other medication except iron, folic acid, vitamins, calcium tablets and DHA since pregnancy? | □0= None, then skip to **F**  □1= Yes, specified________□Unclear |
| E18 | Reasons for medication (multiple choices) | □1= Cold □2= Diabetes □3= Hypertension □4= Heart diseases  □5= Hepatitis B □6= Gastritis □7= Gastric or duodenal ulcer  □8= Kidney diseases □9= Thyroid diseases □10= depression  □11= Others, specified________ |
| **F Have you been diagnosed with the following diseases before this pregnancy by clinicians?** | | |
| F1 | Have you been diagnosed with haematological diseases? (multiple choices) | □0= None □1= Iron deficiency anaemia □2= Megaloblastic anaemia  □3= Aplastic anaemia □4= Thalassemia □5= Unclear  □6= Others, specified_____________ |
| F2 | Have you been diagnosed with hepatitis B infection (HBV)? | □0= None □1=Yes (□2= Carrie, only Hepatitis B surface antigen positive  □3= HBsAg(+), HBeAg (+), HBcAg (+), HBeAg(+), HBeAb(-)  □4= HBsAg(+), HBeAg (+), HBcAg (+), HBeAg(-), HBeAb(+) □5= Unclear) |
| F3 | Have you been diagnosed with hepatitis C (HCV)? | □0= None □1= Yes |
| F4 | Have you been diagnosed with cardiovascular diseases? (multiple choices) | □0= None □1= Chronic hypertension □2= Rheumatic heart disease  □3= Congenital heart disease □4= Cardiomyopathy □5= Hypertensive heart disease □6= Unclear □7= Others, specified _______ |
| F5 | Have you been diagnosed with gynaecological diseases? (multiple choices) | □0= None □1= Hysteromyoma □2= Ovarian cyst  □3= Cervicitis or cervical erosion □4= Polycystic ovary syndrome  □5= Adenomyosis □6= Unclear □7= Others, specified_____________ |
| F6 | Have you been diagnosed with respiratory diseases? (multiple choices) | □0= None □1= Bronchial asthma □2= Tuberculosis  □3= Thoracic deformity □4=Unclear □5= Others, specified_______ |
| F7 | Have you been diagnosed with type 1/2 diabetes? | □0= None □1= Type 1 diabetes □2= Type 2 diabetes  □3= Unclear □4= Others, specified_______ |
| F8 | Have you been diagnosed with thyroid diseases? (multiple choices) | □0= None □1= Hyperthyroidism □2= Hypothyroidism  □3= Subclinical hypothyroidism (hypothyroidism) □4=Unclear  □5= Others, specified_______ |
| F9 | Have you been diagnosed with autoimmune diseases? (multiple choices) | □0= None □1= Systemic lupus erythematosus  □2= Antiphospholipid antibody syndrome □3= Sjogren's syndrome  □4=Unclear □5= Others, specified _______ |
| F10 | Have you been diagnosed with sexually transmitted diseases? (multiple choices) | □0= None □1= Syphilis □2= AIDS (HIV)  □3= Gonorrhoea □4= Condyloma acuminatum □5= Genital herpes  □6= Unclear □7= Others, specified_______ |
| F11 | Have you been diagnosed with digestive system diseases? (multiple choices) | □0= None □1= Chronic gastritis □2= Gastric or duodenal ulcer  □3= Appendicitis □4= Cholecystitis □5= Chronic diarrhea  □6= Haemorrhoids □7= Unclear □8= Ohers, specified_______ |
| F12 | Have you been diagnosed with urinary system disease? (multiple choices) | □0= None □1= Chronic glomerulonephritis □2= Nephrotic syndrome  □3= Chronic renal insufficiency □4= Urinary calculi  □5= Unclear □6= Others, specified_______ |
| F13 | Have you been diagnosed with neurological or mental diseases? (multiple choices) | □0= None □1= Depression □2= Anxiety □3= Epilepsy  □4= Migraine □5=Unclear □6= Others, specified_____________ |

**问卷编号：□□（大区编码）□□（医院编码）□□□□（个人编码）**

**表2 中国孕妇铁缺乏研究调查问卷**

**（孕妇自填）**

**填表说明**：

为了了解您怀孕期间的健康状况，更好地保障您及新生儿的健康，我们将对您怀孕期间的相关状况和部分个人信息进行调查。请您如实回答以下问题，我们保证您所填写的所有信息将仅用于辅助临床治疗和研究目的。除特殊说明外表中所有问题均为单选。请按照实际情况**在合适的选项上划一个勾（√）或填入相关内容，答案没有对错之分**。

| A1 | 姓名{(Name)} | ________ |
| --- | --- | --- |
| A2 | 年龄{(Age)} | □□岁 |
| A3 | 联系电话{(Tel)} | □□□□□□□□□□□(请填写13位手机号) |
| A4 | 就诊卡号{(Card)} | ________ |
| A5 | 末次月经{(LMP)} | □□□□年□□月□□日 □0=**不清楚** |
| A6 | 民族{(Race)} | □0=汉族□1=少数民族：_____族 |
| A7 | 文化程度{(Education)} | □1=研究生及以上 □2=大专及本科□3=中专及高中  □4=初中及小学□5=文盲 |
| A8 | 职业{(Job)} | □1 = 农业/林业/牧业/渔业 (如农民、猎人、牧民、渔民等)  □2 = 生产/运输/设备操作人员（如司机、操作工、电焊工等）  □3 = 服务业人员（如厨师、服务员、理发员、售货员等）  □4 = 办公室人员（如秘书、银行职员、办事员等）  □5 = 专业技术人员 (如医护人员、老师、律师、建筑师、IT、编辑、运动员等)  □6 = 管理人员（政府官员、厂长、经理、行政干部等）  □7 = 未工作  □8 = 其他 |
| A9 | 婚姻状况{(Marriage)} | □1=已婚 □2=未婚□3=丧偶 □4=离异 |
| A10 | 丈夫年龄{(H_age)} | □□岁 |
| A13 | 当地居民（居住半年以上）{(Residence)} | □0=否 □1=是 |
| A14 | 户籍类别{(Hometown)} | □0=城市户口□1=农村户口 |
| A15 | 家庭人数{(Family)} | _____人 |
| A16 | 家庭年收入{(Income)} | □1=3万以下 □2=3-8万（不含8万）  □3=8-12万（不含12万） □4=12-20万（不含20万）  □5=20-30万（不含30万）□6=30万及以上 |
| B1 | 身高{(Height)} | □□□cm |
| B2 | 您**怀孕前**体重{(Weight1)} | □□□kg |
| B3 | 您**目前**体重{(Weight2)} | □□□kg |
| C1 | 全素饮食{(Diet)} | □0=否□1=是 |
| C3 | 您怀孕以来是否有以下生活习惯（多选）{(Habit)} | □0=无 □1=吸烟（平均每日1支以上）  □2=嗜酒（平均每日50ml以上）□3=吸毒 |
| C4 | 您怀孕以来**平均**每天喝牛奶的量{(Milk)} | □1=基本不喝牛奶□2=少于100毫升 □3=100-300毫升（不含300毫升） □4=300-500毫升（不含500毫升） □5=500毫升及以上 |
| C5 | 红肉（猪、牛、羊肉等）摄入量{(Meat)} | 您**怀孕以来平均**每周吃红肉_____次{(Meat_time)}，一次大约____**两**{(Meat)} |
| C6 | 鸡蛋摄入量{(Egg)} | 您**怀孕以来平均**每周吃鸡蛋_____次{(Egg_time)}，一次大约____**克（小鸡蛋约50克/个，大鸡蛋约80克/个）**{(Egg)} |
| C7 | 与您生活在一起的家人或身边的朋友/同事是否吸烟{(Cigarette)} | （平均每日1支以上） □0=否□1=是 |
| D1 | 上次妊娠时间{(Lgestation)} | □1=首次妊娠 □2=距现在半年以内  □3=距现在半年-1年（不含1年） □4=距现在1-2年（不含2年）  □5=距现在2-3年（不含3年） □6=距现在3-4年（不含4年）  □7=距现在4年及以上 |
| D4 | 您是否出现早孕反应（恶心、呕吐、食欲减退）{(Preaction)} | □0=无  □1=轻微（有症状，但不影响日常生活或情绪）  □2=严重（有症状，且影响日常生活或情绪） |
| D8 | 在最近1月内，您是否出现以下症状（多选）{(Syptom)} | □0=无□1=脸色苍白 □2=乏力 □3=心悸  □4=头晕 □5=呼吸困难□6=疲劳  □7=烦躁 □8=易怒□9=注意力下降□10=脱发 |
| E1 | 您怀孕后是否**被医生诊断过缺铁性贫血**{(IDA)} | □0=无 □1=孕早期诊断过（孕1-3月）  □2=孕中期诊断过（孕4-7月）□3=孕晚期诊断过（孕8-10月） |
| E2 | 您**怀孕后**是否服用过铁剂{(Iron1)} | □0=无 □1=力蜚能 □2=速力菲 □3=生血宁 □4=其他铁剂（请注明：________） |
| E3 | 您**现在**是否正在服用铁剂{(Iron2)} | □0=无(**选”无”跳转E5**)□1=力蜚能 □2=速力菲  □3=生血宁 □4=其他铁剂（请注明：________） |
| E4 | 您已经服用铁剂多长时间{(Itime)} | □1=1个月以内 □2=1-3个月（不含3个月）  □3=3-6个月（不含6个月） □4=6个月及以上 |
| E5 | 您有无补充叶酸{(Folic)} | □0=无(**选”无”跳转E9**)□1=有 |
| E6 | 开始补充叶酸时间{(Ftime1)} | □1=孕前 □2=孕1-3月  □3=孕4-7月 □4=孕8-10月 |
| E7 | 补充叶酸**持续**时间{(Ftime2)} | □1=1个月以内 □2=1-3个月（不含3个月）  □3=3-6个月（不含6个月） □4=6个月及以上 |
| E8 | 服用叶酸剂量{(Fdose)} | ______片/天；______天/每周 |
| E9 | 您有无服用复合维生素{(Vitamin)} | □0=无(**选”无”跳转E13**)□1=爱乐维  □2=玛特纳 □3=其他维生素（请注明：________） |
| E10 | 开始服用复合维生素时间{(Vtime1)} | □1=孕前 □2=孕1-3月  □3=孕4-7月 □4=孕8-10月 |
| E11 | 服用复合维生素**持续**时间{(Vtime2)} | □1=1个月以内 □2=1-3个月（不含3个月）  □3=3-6个月（不含6个月） □4=6个月及以上 |
| E12 | 服用复合维生素剂量{(Vdose)} | ______片/天；______天/每周 |
| E13 | 您有无补钙{(Calcium)} | □0=无(**选”无”跳转E17**)□1=钙尔奇  □2=迪巧□3=其他钙片（请注明：________） |
| E14 | 开始补钙时间{(Ctime1)} | □1=孕前 □2=孕1-3月  □3=孕4-7月 □4=孕8-10月 |
| E15 | 补钙持续时间{(Ctime2)} | □1=1个月以内 □2=1-3个月（不含3个月）  □3=3-6个月（不含6个月） □4=6个月及以上 |
| E16 | 服用钙片剂量{(Cdose)} | _____片/天 ；______天/每周 |
| E17 | 您怀孕以来是否服用过除铁剂、叶酸、维生素、钙片、DHA以外的药物{(Odrug)} | □0=无（**跳至F表**）  □1=有（请注明药名：________□不记得） |
| E18 | 您服药的原因（可多选）{(Dreason)} | □1=感冒 □2=糖尿病 □3=高血压  □4=心脏病 □5=乙肝 □6=胃炎  □7=胃/十二指肠溃疡 □8=肾病 □9=甲状腺疾病  □10=抑郁症 □11=其它（请注明：________） |
| **F 请回忆您在怀孕前是否患有经医生诊断的以下疾病**： | | |
| F1 | 您是否患有血液系统疾病{(Hematopath)} | □0=无 □1=缺铁性贫血 □2=巨幼细胞贫血 □3=再生障碍性贫血 □4=地中海贫血□5=不清楚 □6=其它（请说明：_______） |
| F2 | 您是否患有乙肝{(HBV)} | □0=无 □1=有（□2=携带者，仅表面抗原阳性 □3=大三阳 □4=小三阳 □5=不清楚类型） |
| F3 | 您是否患有丙肝{(HCV)} | □0=无 □1=有 |
| F4 | 您是否患有心血管疾病(多选){(Cardiopath)} | □0=无 □1=慢性高血压 □2=风湿性心脏病  □3=先天性心脏病 □4=心肌病 □5=高血压性心脏病  □6=不清楚 □7=其它（请说明：_______） |
| F5 | 您是否患有妇科疾病(多选){(Gynopathy)} | □0=无 □1=子宫肌瘤□2=卵巢囊肿  □3=宫颈炎/宫颈糜烂□4=多囊卵巢综合征 □5=子宫腺肌症  □6=不清楚 □7=其它（请说明：_______） |
| F6 | 您是否患有呼吸系统疾病(多选){(Respdis)} | □0=无 □1=支气管哮喘 □2=肺结核  □3=胸廓畸形 □4=不清楚 □5=其它（请说明：_______） |
| F7 | 您是否患有糖尿病**（非妊娠糖尿病）**{(DM)} | □0=无 □1=1型糖尿病□2=2型糖尿病  □3=不清楚□4=其它（请说明：_______） |
| F8 | 您是否患有甲状腺疾病(多选){(Thyropathy)} | □0=无 □1=甲状腺功能亢进症□2=甲状腺功能减退症□3=亚临床甲状腺功能减退（亚[甲减](http://baike.baidu.com/view/99858.htm)）□4=不清楚  □5=其它（请说明：_______） |
| F9 | 您是否患有免疫系统疾病(多选) {(Immune)} | □0=无 □1=系统性红斑狼疮□2=抗磷脂抗体综合征 □3=干燥综合征 □4=不清楚 □5=其它（请说明：_______） |
| F10 | 您是否患有性传播疾病(多选){(STD)} | □0=无 □1=梅毒 □2=艾滋病（HIV）  □3=淋病 □4=尖锐湿疣 □5=生殖器疱疹  □6=不清楚 □7=其它（请说明：_______） |
| F11 | 您是否患有消化系统疾病(多选) {(Digestive)} | □0=无 □1=慢性胃炎 □2=胃/十二指肠溃疡  □3=阑尾炎 □4=胆囊炎 □5=慢性腹泻  □6=痔疮 □7=不清楚 □8=其它（请说明：_______） |
| F12 | 您是否患有泌尿系统疾病(多选) {(Urinary)} | □0=无 □1=慢性肾小球肾炎□2=肾病综合征  □3=慢性肾功能不全□4=尿路结石□5=不清楚  □6=其它（请说明：_______） |
| F13 | 您是否患有神经或精神疾病(多选){(Neupsypath)} | □0=无 □1=抑郁症 □2=焦虑症  □3=癫痫 □4=偏头疼 □5=不清楚  □6=其它（请说明：_______） |

**Table 3 Survey on iron deficiency among pregnant women in China**

**(Doctor Investigation)**

**Survey Number: □□Region Code □□ Hospital Code □□□□ Person Code**

Mother's Name _______ Visit card number ________Telephone number________

| **G Gestational and visiting information** | | |
| --- | --- | --- |
| G1 | Blood routine test | RBC______*10^12^/L HGB______g/L HCT______ WBC______*10^9^/L  NEUT (Neutrophil count) ______*10^9^/L PLT______*10^12^/L  Test date □□□□Year□□Month□□Day |
| G2 | Serum ferritin | ______ng/ml Test date □□□□Year □□Month □□Day |
| G3 | Visiting date | □□□□Year □□Month □□Day |
| G4 | Doctor's name |  |
| G5 | Gestational week | □Week □Day |
| G6 | Gravidity | □Times |
| G7 | Parity (gestational week more than 28 weeks except this time) | □Times |
| G8 | Induced abortion | □Times |
| G9 | Spontaneous abortion | □Times |
| G10 | Caesarean section history | □0= No □1= Yes |
| G11 | Use of assisted reproductive technology (ART) | □0=None, then skip to **G13** □1= Yes |
| G12 | Methods of assisted reproductive technology | □1= In vitro fertilization and embryo transfer (IVF-ET)  □2= Intrauterine insemination (IUI) □3= Others, specified______ |
| G13 | Number of pregnant babies | □1= singleton □2= Twins □3= Triplets or more |
| G14 | Weight at visiting this time | □□□.□kg |
| G15 | Blood pression this visit | Systolic blood pressure (SBP) □□□mmHg  Diastolic blood pressure (DBP) □□□mmHg |
| G16 | Iron deficiency diagnosis (ID) | □0= No, then skip to **H0** □1= Yes |
| G17 | Iron deficiency anaemia diagnosis (ID) | □0= No □1= Yes |
| G17.1 | Diagnosis timing for Iron deficiency anaemia | □0=None □1= Gestational 1-3 months  □2= Gestational 4-7 months □3= Gestational 8-10 months |
| G18 | Treatment project for iron deficiency | □0= None, then skip to **H0** □1= Oral iron □2= Injection iron  □3= Diet improvement □4= Oral iron + Diet improvement  □5= Injection iron + Diet improvement □6= Blood transfusion  □7= Others, specified______ |
| G19 | Types of iron supplementation prescribed by doctors (multiple choices) | □1= Polysaccharide iron complex (LI-FEI-ENNG)  □2= Ferrous succinate (sulpiride) (SU-LI-FEI)  □3= SHENG-XUE-NING □4= Ferric dextran oral liquid  □5= Ferrous fumarate □6= Ferrous sulphate  □7= Ferrous sulfate controlled-release tablets (FU-NAI-DE)  □8= Ferrous gluconate □9= Protein succinic acid oral solution  □10= Iron gluconate injection □11= Sorbitol iron injection  □12= Iron dextran injection □13= Sucrose iron injection  □14= Others, specified______ |
| G20 | Iron supplement dose | Oral______ pieces per day; Injection ______ mg per day |
| G21 | Iron supplement time | □1= 1 month □2= 2 months □3= 3 months □4= >3 months □5= Unclear |
| **H Pregnancy complications (multiple choices)** | | |
| H0 | Pregnancy complications | □0= None, skip to **I** □1= Yes |
| H1 | Haematological diseases (multiple choices) | □0= None □1= Iron deficiency anaemia □2= Megaloblastic anaemia  □3= Aplastic anaemia □4= Thalassemia |
| H2 | Hepatitis B infection (HBV) diagnosed during first trimester | □0= None □1= Yes (□HBsAg □HBsAb □HBeAg □HBeAb □HBcAb) |
| H3 | Hepatitis B DNA test result | □ <10^3^ copies/ml □10^3^-10^5^ copies/ml □＞10^5^ copies/ml □No test |
| H4 | Hepatitis C infection (HCV) diagnosed during first trimester | □0= None □1= Yes ( Anti-HCV positive ) |
| H5 | Cardiovascular diseases (multiple choices) | □0= None □1= Chronic hypertension □2= Rheumatic heart disease  □3= Congenital heart disease □4= Cardiomyopathy □5= Hypertensive heart disease □6= Others, specified _______ |
| H6 | Gynaecological diseases (multiple choices) | □0= None □1= Hysteromyoma □2= Ovarian cyst  □3= Cervicitis or cervical erosion □4= Polycystic ovary syndrome  □5= Adenomyosis □6= Others, specified_____________ |
| H7 | Respiratory diseases (multiple choices) | □0= None □1= Bronchial asthma □2= Tuberculosis  □3= Thoracic deformity □4= Others, specified_______ |
| H8 | Diabetes (not including gestational diabetes) | □0= None □1= Type 1 diabetes □2= Type 2 diabetes |
| H9 | Thyroid diseases (multiple choices) | □0= None □1= Hyperthyroidism □2= Hypothyroidism  □3= Subclinical hypothyroidism (hypothyroidism) □4= Others, specified_______ |
| H10 | Autoimmune diseases (multiple choices) | □0= None □1= Systemic lupus erythematosus  □2= Antiphospholipid antibody syndrome □3= Sjogren's syndrome  □4= Others, specified _______ |
| H11 | Sexually transmitted diseases (multiple choices) | □0= None □1= Syphilis □2= AIDS (HIV) □3= Gonorrhoea  □4= Condyloma acuminatum □5= Genital herpes □6= Others, specified_____ |
| H12 | Digestive system diseases (multiple choices) | □0= None □1= Chronic gastritis □2= Gastric or duodenal ulcer  □3= Appendicitis □4= Cholecystitis □5= Chronic diarrhea  □6= Haemorrhoids □7= Ohers, specified_______ |
| H13 | Urinary system diseases (multiple choices) | □0= None □1= Chronic glomerulonephritis □2= Nephrotic syndrome  □3= Chronic renal insufficiency □4= Urinary calculi  □5= Others, specified_______ |
| H14 | Neurological or mental diseases (multiple choices) | □0=0= None □1= Depression □2= Anxiety □3= Epilepsy  □4= Migraine □5= Others, specified___________ |
| J | Additional information to be specified | □0=None □1= Yes, specified_____________________________ |

**表3 中国孕妇铁缺乏流行病学研究横断面调查**

**（医生填写问卷）**

孕妇姓名{(Name)}：_______ 就诊卡号{(Card)}：________ 手机号{(Tel)}：________

**问卷编号：□□（大区编码）□□（医院编码）□□□□（个人编码）**

| **G** | **本次妊娠及就诊信息** | |
| --- | --- | --- |
| G1 | 血常规{(Bloodroutine)} | RBC______*10^12^/L HGB______g/LHCT______ WBC______*10^9^/L NEUT（中性粒细胞计数）______*10^9^/L  PLT______*10^12^/L  检查日期{(Blood_date)}：□□□□年□□月□□日 |
| G2 | 血清铁蛋白{(Ferritin)} | ______ng/ml检查日期{(Fer_date)}：□□□□年□□月□□日 |
| G3 | 就诊日期{(Date)} | □□□□年□□月□□日 |
| G4 | 就诊医生姓名{(Doctor)} |  |
| G5 | 就诊时孕周{(Gweek)} | □周□天（核实后孕周） |
| G6 | 孕次{(Gravidity)} | □次 |
| G7 | 产次{(Parity)} | □次（除外本次分娩的所有≥28周分娩的次数） |
| G8 | 人工流产{(Iabortion)} | □次 |
| G9 | 自然流产{(Nabortion)} | □次 |
| G10 | 剖宫产史{(Cesarean)} | □0=无□1=有 |
| G11 | 采用辅助生育技术{(ART)} | □0=无（**跳至G13**）□1=有 |
| G12 | 辅助生育所用技术{(Artdetails)} | □1=体外受精与胚胎移植（IVF-ET）□2=宫腔内人工受精（IUI）  □3=其他 |
| G13 | 胎数{(Embryo)} | □1=单胎 □2=双胎 □3=三胎及以上 |
| G14 | 本次就诊体重{(Weight3)} | □□□.□kg |
| G15 | 本次就诊血压 | 收缩压{(SBP)}□□□mmHg 舒张压{(DBP)}□□□mmHg |
| G16 | 诊断铁缺乏{(ID)} | □0=无（**跳至H0**）□1=有 |
| G17 | 诊断缺铁性贫血{(IDA1)} | □0=无 □1=有 |
| G17.1 | 诊断缺铁性贫血时间{(IDA_Time)} | □0=无 □1=孕早期诊断过（孕1-3月）  □2=孕中期诊断过（孕4-7月）□3=孕晚期诊断过（孕8-10月） |
| G18 | 铁缺乏治疗方案{(Project)} | □0=无（**跳至H0**）□1=口服铁剂□2=注射铁剂  □3=改善饮食 □4=口服铁剂+改善饮食 □5=注射铁剂+改善饮食□6=输血□7=其他 |
| G19 | 医嘱铁制剂种类**(可多选)**{(Iron)} | □1=多糖铁复合物（力蜚能） □2=琥珀酸亚铁（速力菲）  □3=生血宁 □4=右旋糖酐铁口服液  □5=富马酸亚铁□6=硫酸亚铁  □7=硫酸亚铁控释片（福乃得） □8=葡萄糖酸亚铁  □9=蛋白琥珀酸口服溶液□10=葡萄糖酸铁纳注射液  □11=山梨醇铁注射液 □12=右旋糖酐铁注射液  □13=蔗糖铁注射液 □14=其他铁剂 |
| G20 | 医嘱补铁剂量{(I_dose)} | 口服______片/天**和/或**注射______mg/天 |
| G21 | 服用时间{(I_time)} | □1=1个月 □2=2个月 □3=3个月 □4=3个月以上  □5=不清楚 |
| **H** | **基础疾病（妊娠合并症，可多选）** | |
| H0 | 妊娠合并症{(Diseases)} | □0=无 □1=有**（若"无"则跳至I表）请确定有无以下合并症** |
| H1 | 血液系统疾病{(Hematopath1)} | □0=无  □1=缺铁性贫血□2=巨幼细胞贫血  □3=再生障碍性贫血 □4=地中海贫血 |
| H2 | 乙肝（孕早期检测）{(HBV1)} | □0=无  □1=有（□HBsAg □HBsAb □HBeAg □HBeAb □HBcAb） |
| H3 | 乙肝DNA检测结果{(HBVDNA)} | □＜10^3^copies/ml□10^3^-10^5^copies/ml□＞10^5^copies/ml  □未测 |
| H4 | 丙肝（孕早期检测）{(HCV1)} | □0=无 □1=有(抗-HCV阳性) |
| H5 | 心血管疾病{(Cardiopath1)} | □0=无 □1=慢性高血压 □2=风湿性心脏病  □3=先天性心脏病 □4=心肌病 □5=高血压性心脏病  □6=其它（请说明：_______） |
| H6 | 妇科疾病{(Gynopathy1)} | □0=无 □1=子宫肌瘤□2=卵巢囊肿  □3=宫颈炎/宫颈糜烂□4=多囊卵巢综合征 □5=子宫腺肌症  □6=其它（请说明：_______） |
| H7 | 呼吸系统疾病{(Respdis1)} | □0=无 □1=支气管哮喘 □2=肺结核  □3=胸廓畸形 □4=其它（请说明：_______） |
| H8 | 糖尿病**（非妊娠糖尿病）{(DM1)}** | □0=无 □1=1型糖尿病□2=2型糖尿病 |
| H9 | 甲状腺疾病{(Thyropathy1)} | □0=无 □1=甲状腺功能亢进症□2=甲状腺功能减退症  □3=亚甲减 |
| H10 | 免疫系统疾病{(Immune1)} | □0=无 □1=系统性红斑狼疮□2=抗磷脂抗体综合征 □3=干燥综合征 |
| H11 | 性传播疾病{(STD1)} | □0=无 □1=梅毒 □2=艾滋病（HIV）  □3=淋病 □4=尖锐湿疣 □5=生殖器疱疹  □6=其它（请说明：_______） |
| H12 | 消化系统疾病{(Digestive1)} | □0=无 □1=慢性胃炎 □2=胃/十二指肠溃疡  □3=阑尾炎 □4=胆囊炎 □5=慢性腹泻  □6=痔疮 □7=其它（请说明：_______） |
| H13 | 泌尿系统疾病{(Urinary1)} | □0=无 □1=慢性肾小球肾炎□2=肾病综合征  □3=慢性肾功能不全□4=其它（请说明：_______） |
| H14 | 神经或精神疾病{(Neupsypath1)} | □0=无 □1=抑郁症 □2=焦虑症  □3=癫痫 □4=偏头疼 □5=其它（请说明：_______） |
| J | 该问卷(包括孕妇自填和医生填写部分)是否有其他需要说明的问题{(Suppl)} | □0=无 □1=有 （请注明：_____________________________） |
